# Colorectal Adenocarcinoma Metastasis to the Hard Palate: An Uncommon Site of Metastasis for a Common Malignancy

**DOI:** 10.7759/cureus.33082

**Published:** 2022-12-29

**Authors:** Sandhya Kolagatla, Rewanth Katamreddy, Jonathan Piercy, Subramanya shyam Ganti, Nagabhishek Moka

**Affiliations:** 1 Internal Medicine, Appalachian Regional Healthcare, Whitesburg, USA; 2 Internal Medicine, St. Michael Medical Center, Newark, USA; 3 Internal Medicine/Pulmonary Critical Care, Appalachian Regional Healthcare, Harlan, USA; 4 Oncology/Hematology/Internal Medicine, Appalachian Regional Healthcare, Hazard, USA

**Keywords:** adenocarcinoma of colon, oral biopsy, occult metastases, oral potentially malignant lesion, oral cavity metastasis, hard palate, colorectal cancer

## Abstract

Colorectal adenocarcinoma (CRC) most commonly metastasizes to the peritoneum, liver, lung, and bone. Metastasis to the oral cavity is uncommon. Here, we report the case of a 74-year-old man who presented with a few months of chewing and swallowing difficulty, shoulder pain, and weight loss of 30 pounds. On oral exam, he was noted to have a 5 cm fixed hard palate mass. Primary hard palate malignancy was initially suspected. Biopsy of the mass confirmed adenocarcinoma with an immunohistochemical pattern suggestive of colorectal origin. He was later found to have extensive skeletal metastasis. Palliative radiotherapy to the hard palate region was initiated, followed by palliative systemic chemotherapy. We have found only three other published cases of rectal adenocarcinoma with hard palate metastasis.

## Introduction

Metastasis from distant primary tumors comprises 1-3% of oral region malignancies. The most common primary tumors that metastasize to the oral mucosa are lung, liver, breast, and kidney cancers. Metastasis to the oral region can involve bony or soft tissues. The mandible is a common site for bony oral metastasis, though the gingiva is the most common site, and the tongue is the second most common site for soft tissue oral metastasis. Metastasis to the oral region can be the initial presentation of an undiagnosed malignancy [1].

The most common sites for colorectal adenocarcinoma (CRC) metastasis are the liver, lungs, and bones [2]. CRC metastasis to the oral cavity occurs in less than 1% of cases. We have found only a few case reports published of CRC metastasis to the hard palate [3-5]. In this case report, we present a patient with a hard palate mass as an initial presentation of metastatic colorectal adenocarcinoma.

## Case presentation

A 74-year-old male presented to the office with right shoulder pain and trouble chewing and swallowing for the preceding few months. Past medical history was significant for stage IIIb rectal adenocarcinoma treated with concurrent chemotherapy and radiation followed by further chemotherapy and surgery three years back. He had a total 50-pack-a-year history of smoking but quit five years earlier. A review of systems revealed a 30-pound weight loss and generalized musculoskeletal pain.

**Figure 1:**
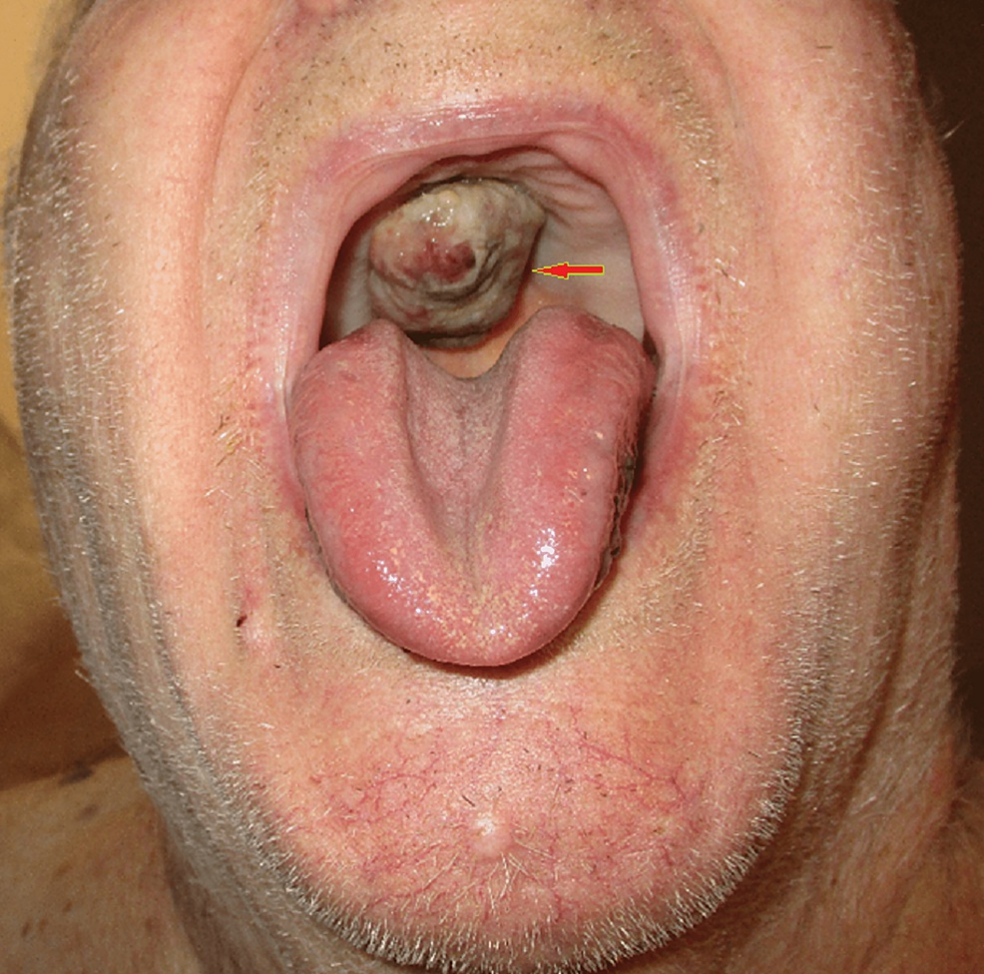
Oral cavity exam: protruding and hard mass (red arrow) arising from roof of the mouth

Examination of his oral cavity revealed a protruding, 5 cm diameter mass in the roof of his mouth on the palate (Figure 1).

**Figure 2:**
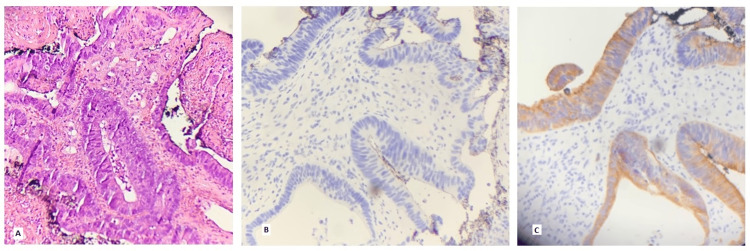
Hard palate mass core biopsy histopathology: (A) Hematoxylin and eosin stain demonstrating tumor cells in glandular formation (adenocarcinoma) with tumor necrosis; (B) Tumor cells negative for cytokeratin (CK) 7 stain; (C) Tumor cells positive for CK 20 stain.

Biopsy of the hard palate mass revealed adenocarcinoma with extensive tumor necrosis (Figure 2A), with immunohistochemical staining of cytokeratin 7 negative (Figure 2B) and cytokeratin 20 positive (Figure 2C).

**Figure 3:**
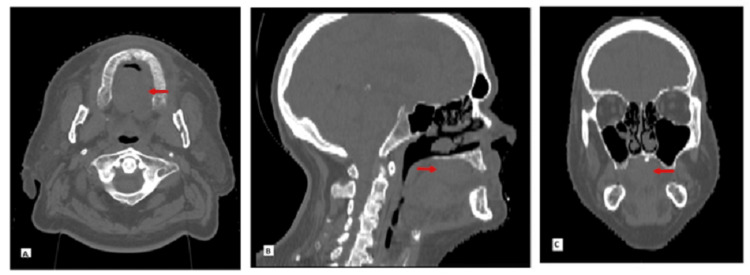
Non-contrast CT of sinuses and face: (A) Transverse section, (B) Sagittal section, and (C) Coronal section demonstrating hard palate mass (red arrow) with bony destruction

Next-generation sequencing was positive for KRAS oncogene mutation and negative for BRAF and microsatellite instability. Endoscopy and colonoscopy did not reveal intraluminal malignancy. A computed tomography scan showed a 5 cm hard palate mass with bony destruction (Figure 3).

**Figure 4:**
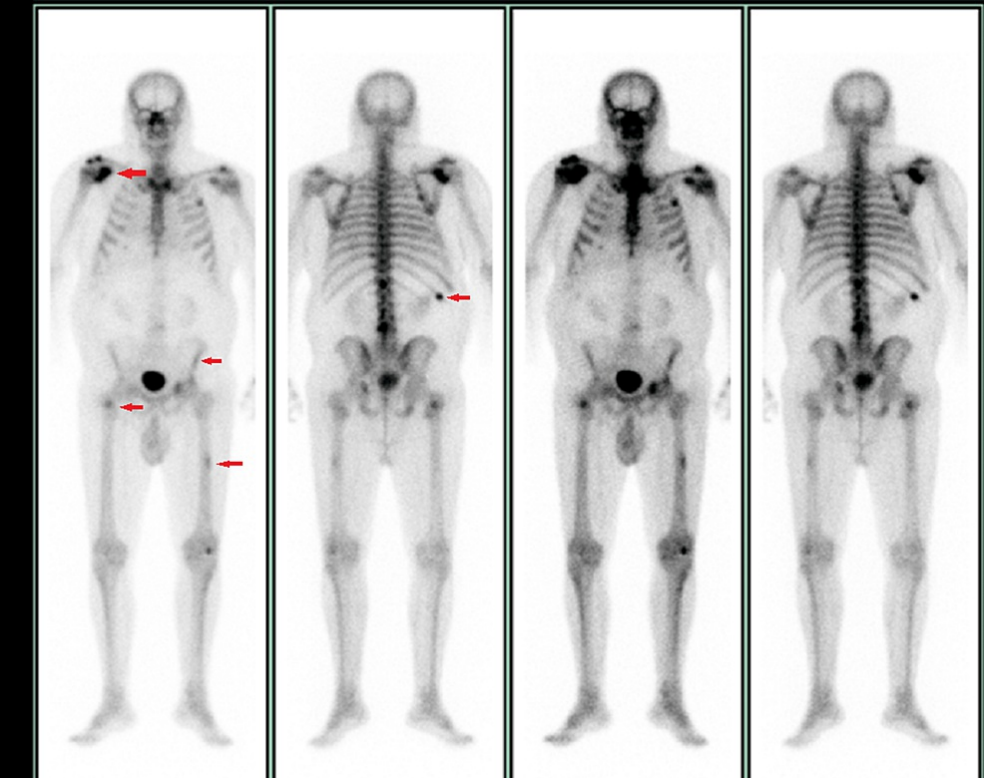
Three-phase whole-body technetium bone scan demonstrating increased uptake in right proximal humerus, scapula, right proximal femur, left mid femur, left acetabulum, and right 10th rib (red arrows).

A three-phase whole-body technetium bone scan showed increased uptake in the right proximal humerus, scapula, right proximal femur, left mid femur, left acetabulum, and right 10th rib that was suspicious for metastasis (Figure 4). 

A percutaneous endoscopic gastrostomy (PEG) tube was necessary to provide nutrition and medication. He completed palliative radiation therapy of 2100 centigray (cGy) to the hard palate mass. He was started on 5-fluorouracil (bolus of 400 mg/m2 IV, infusional dose of 2400 mg/m2 IV), leucovorin calcium (calcium folinate) 400 mg/m2 IV, irinotecan 180 mg/m2 IV (FOLFIRI), and bevacizumab 5 mg/kg IV every 14 days. He tolerated chemotherapy well, except for anticipated diarrhea, which was controlled with loperamide, as needed. Over the next few weeks, his oral symptoms improved, and he was able to increase his oral intake. He declined any further treatment for personal reasons. He succumbed within six months of diagnosis.

## Discussion

Colorectal adenocarcinoma most commonly occurs in patients older than 60 years. It is the third most common carcinoma in the world [6]. Approximately 20% of patients with CRC have metastasis on presentation [7]. Oral metastasis is not a common site for the deposition of metastatic cells.

The symptoms of oral metastasis vary based on their exact location. Oral metastasis commonly presents with swelling, dysphagia, dysarthria, odynophagia, and bleeding similar to primary oral malignancies [8]. Metastatic lesions to the oral cavity can be misdiagnosed as benign lesions or primary oral squamous cell carcinoma [9]. Metastatic deposits to the oral cavity are usually not high on the list of differential diagnoses of an oral lesion; as such, diagnosis of the occult malignancy is often delayed. Biopsy of the oral lesion is the gold standard for making the diagnosis.

When a metastatic deposit in the oral cavity is suspected, computed tomography of the chest, abdomen, and pelvis can be used to identify the occult primary malignancy. Positron electron transmission (PET) scanning with fluorodeoxyglucose (FDG) can be considered for specific cases of unknown primary [10]. Serum tumor markers can be helpful in certain cases to identify the primary malignancy but should be obtained in the right clinical setting because of lack of specificity [11]. 

The most common immunohistochemistry (IHC) pattern for CRC is positive for CK 20 and negative CK 7, but the pattern can be variable [12]. Autopsy studies demonstrated the histological subtype of CRC as a predictive variable for the metastatic site, although the mechanism is not well understood [13]

Treatment for oral metastases depends on the primary tumor type and the extent of tumor spread. A substantial majority of oral metastatic lesions are part of the extensive spread of malignancy, in which case systemic therapies must be pursued. Palliative radiotherapy for metastatic oral lesions can help alleviate local symptoms [14].

Prognosis is generally poor with CRC oral metastasis with an average survival of seven months in a retrospective study [1]. Longer survival is possible when the metastatic lesion is isolated and can be approached surgically. Early nutritional assessment and individually tailored support are key in patients with either primary or metastatic oral lesions [15].

## Conclusions

In this case, a hard palate mass was the initial presentation of widespread metastatic CRC. Although this is a rare finding, metastatic tumor deposits from an occult malignancy must be considered when evaluating a palate mass. Histopathological and relevant imaging studies must be considered to evaluate distant primary malignancies. Treatment of these findings depends on the primary tumor type and the extent of the metastasis, and should include nutritional and symptomatic support.
